# Further Evidence That the Effects of Repetition on Subjective Time Depend on Repetition Probability

**DOI:** 10.3389/fpsyg.2017.01915

**Published:** 2017-11-01

**Authors:** William J. Skylark, Ana I. Gheorghiu

**Affiliations:** ^1^Department of Psychology, University of Cambridge, Cambridge, United Kingdom; ^2^Department of Psychology, University of Essex, Colchester, United Kingdom

**Keywords:** perception, time perception, expectation, repetition suppression, predictive coding

## Abstract

Repeated stimuli typically have shorter apparent duration than novel stimuli. Most explanations for this effect have attributed it to the repeated stimuli being more expected or predictable than the novel items, but an emerging body of work suggests that repetition and expectation exert distinct effects on time perception. The present experiment replicated a recent study in which the probability of repetition was varied between blocks of trials. As in the previous work, the repetition effect was smaller when repeats were common (and therefore more expected) than when they were rare. These results add to growing evidence that, contrary to traditional accounts, expectation increases apparent duration whereas repetition compresses subjective time, perhaps via a low-level process like adaptation. These opposing processes can be seen as instances of a more general “processing principle,” according to which subjective time is a function of the perceptual strength of the stimulus representation, and therefore depends on a confluence of “bottom-up” and “top-down” variables.

## Introduction

Stimulus repetition affects the perception of time. Most studies of the effects of repetition on subjective duration have used a “temporal oddball” paradigm, in which participants see or hear a sequence of repeated standard stimuli, with occasional presentations of a different “oddball” item. The oddballs are judged to have longer duration than the standards (Tse et al., 2004). This effect occurs with a wide variety of simple and complex visual and auditory stimuli and with a range of temporal judgment tasks (Tse et al., 2004; Kim and McAuley, 2013; Birngruber et al., 2014), although the effect has sometimes been exaggerated because researchers often choose oddballs that are predominantly shorter in duration than the standards (Seifried and Ulrich, 2010).

The oddball task has several drawbacks: the novelty of the oddball is confounded with its position in the sequence, participants are asked to compare a single oddball duration with the duration of many standards on either side of it (such that the decision process is ill specified), and the task confounds novelty on a given trial with the overall probability of occurrence across all trials (Matthews, 2011; Birngruber et al., 2015a).

A simpler approach involves presenting two stimuli on each trial and having participants indicate whether the second, target stimulus was shown for more or less time than the first (a “reminder task”). On some trials the non-temporal properties of the second stimuli are identical to those of the first (e.g., the same image is repeated); on other trials the second stimulus is novel. This approach de-confounds the target’s status as “novel” or “repeat” from its position in the trial, and only requires people to compare the durations of two items; it also allows the experimenter to present completely new stimuli on each trial, so that the repetition of a stimulus within a trial is not confounded with its frequency of presentation across trials. The data from this task replicate the basic repetition effect found in the oddball task: repeated items are judged to last for less time than novel ones (Matthews, 2011, 2015; Birngruber et al., 2015b).

The basis for repetition effects is unclear. The oddball effect was initially attributed to attentional capture: novel stimuli capture attention and were argued to thereby accelerate the rate of information processing, which serves as an index of the passage of time (Tse et al., 2004). One problem with this idea is that it is the *non-temporal* properties of the oddball that capture attention (e.g., its color or shape), and studies of attentional effects on timing indicate that greater attention to non-temporal features causes subjective time to contract rather than expand – putatively because one “misses” pulses from an internal pacemaker when one is not “attending to time” (e.g., Casini and Macar, 1997; Block et al., 2010). A second proposal is that novel or rare stimuli are more arousing than repeated ones and therefore accelerate the rate of the putative pacemaker (e.g., Ulrich et al., 2006; New and Scholl, 2009; for a recent discussion of related ideas see Wearden et al., 2017). A third suggestion is that subjective duration is positively related to the overall neural response evoked by the stimulus – the “coding efficiency” (Eagleman and Pariyadath, 2009). Repeated items often evoke smaller responses (“repetition suppression”; e.g., Grill-Spector et al., 2006), which in turn is argued to underlie the contraction of subjective duration for repeated items (Pariyadath and Eagleman, 2007, 2012; Schindel et al., 2011; see Matthews et al., 2014, for a review). Finally, it has been suggested that the temporal and non-temporal relations between items generate expectations about future stimuli such that less expected (i.e., novel) stimuli are detected faster and therefore seem to last longer (Kim and McAuley, 2013).

These frameworks share the basic assumption that the repetition effect arises because repeated stimuli are expected whereas novel stimuli are unexpected. That is, they equate repetition with expectation.

To test this core assumption, Matthews (2015) adapted an approach used in studies of neural repetition suppression and in investigations of priming effects (e.g., Bodner and Masson, 2004; Summerfield et al., 2008). In these studies, each trial presents two stimuli and the probability that the second is a repeat of the first varies across blocks: in blocks with a high repetition rate (rep-rate), the second stimulus is usually the same as the first; in blocks with a low rep-rate, the second stimulus is usually novel. The logic of this approach is that, if repetition effects arise because repeated stimuli are more expected than novel ones, then making the repeated stimuli even more predictable ought to increase the strength of the repetition effect. That is, repetition effects will be stronger in high rep-rate blocks than in low rep-rate blocks. This result has been reported in several studies of neural repetition suppression: the evoked response was smaller for repeats than for novel items, and this suppression was greater in blocks where repetition was common (e.g., Summerfield et al., 2008, 2011; Larsson and Smith, 2012; Andics et al., 2013; Mayrhauser et al., 2014).

However, Matthews (2015) found the opposite pattern for timing responses: repeats were judged to last for less time than novel stimuli, but this effect was reduced, eliminated, and even reversed in blocks where the repetition rate was high. This argues against the idea that the effect of repetition on subjective time arises because repeated stimuli are more expected. Rather, they indicate opposing influences of first-order repetition and second-order expectations, with the former causing a compression of subjective duration and the latter an expansion.

Similar results have since been reported by Cai et al. (2015). These authors found that repetition compresses subjective duration even when the repeated item is surprising. For example, the authors presented regular sequences where the last item was a non-repeat (e.g., A-B-A-B-A) and irregular sequences where the last item was unpredictable but repeated the preceding item (e.g., A-B-A-B-B). The last item was judged to have longer duration in the former condition, indicating a compressive effect of repetition that is stronger than any effect of expectation. Like Matthews, Cai et al. (2015) argue that adaptation in the sensory cortices underpins the repetition effect on time perception. In addition, Cai et al. (2015) found that the compressive effect of repetition was more pronounced when repeats were rare (20% of trials), than when they were common (80% of trials) – mirroring the result from Matthews (2015), and indicating that making a repeat more predictable actually offsets the compressive effect of repetition.

A similar conclusion is urged by recent work from Birngruber et al. (2017), which found that explicit expectations about forthcoming stimuli do indeed serve to expand subjective time. These authors asked people to predict the color or shape of stimuli immediately before they were presented; items that matched the prediction were judged to last longer than those that did not.

The present experiment provides a small addition to the body of data regarding the dissociable effects of first-order repetition and higher-order expectations. The experiment replicates the approach of Matthews (2015), and had three aims. The first was simply to generalize the findings of the earlier work by using slightly different stimuli, testing equipment, and participants, and with the data collected by a different researcher. The second was to address a shortcoming in the design of the earlier studies. In those experiments, the duration of the first and second stimuli on each test trial were identical. This was to maximize participants’ uncertainty and thereby to maximize the effects of non-temporal information (repetition and repetition rate). However, this approach raises the possibility that the effect would disappear as soon as there was genuine temporal information that could be used as the basis for the participant’s response; alternatively, the temporal information may interact with the effects of repetition and rep-rate. Finally, the current study increased the number of trials in the total session from that used in previous work. Given the striking dissociation between the effects of rep-rate on time perception and its effects on neural repetition suppression, it will be important to examine both effects simultaneously by collecting neural data whilst participants complete the temporal discrimination task. Such neuroimaging studies require a large number of trials per condition, and the current experiment provides a behavioral prototype for a task that could later be run with participants whose neural responses are recorded by EEG or MEG.

## Materials and Methods

### Ethics Statement

The experiment was approved by the University of Essex Faculty of Biology Research Ethics Committee. Participants provided written informed consent prior to starting the task.

### Stimuli

The stimuli were 873 face images adapted from publicly available face databases described by O’Toole et al. (2005), Ebner (2008), and Burton et al. (2010). All images were edited to be 280 pixels high (widths varied slightly depending on the proportions of the face) and were presented on a uniform gray background). The stimuli were presented on 60 Hz LCD monitors with a resolution of 1920 pixels × 1080 pixels viewed from approximately 50 cm, giving a vertical visual angle of approximately 7.9°.

### Design and Procedure

On each trial participants saw two faces, one after the other. Their task was to judge whether the second face was presented for more or less time than the first; they indicated their judgment with a keypress. On repetition trials, the second face was identical to the first; on non-repetition trials, the second face was novel. The blocks alternated between high rep-rate blocks, where 75% of trials were repetitions and 25% were non-repetitions, and low rep-rate blocks, where 25% were repetitions and 75% were non-repetitions.

The trial structure is shown in **Table 1**. Most trials (40 per block) were test trials, which involved a difficult temporal discrimination: the first face was shown for 567 ms or 633 ms with equal frequency, and the second was shown for 600 ms. The remaining trials were catch trials, designed to detect inattentive participants. On half of the catch trials the first face was shown for 633 ms and the second was shown for 300 ms; on the other half the first face was shown for 567 ms and the second was shown for 1200 ms. The proportion of repetition and non-repetition trials for each duration combination matched the current repetition rate, as shown in **Table 1**. No faces were repeated across trials (i.e., the first face on each trial was always completely novel). Trial order within each block was randomized separately for each participant.

**Table 1:** The trial structure.

Block type	Trial type	Condition	First face duration (ms)	Second face duration (ms)	N trials
Low rep-rate	Test trials	Novel	567	600	15


		Novel	633	600	15
		Repeat	567	600	5
		Repeat	633	600	5
	Catch trials	Novel	567	1200	3


		Novel	633	300	3
		Repeat	567	1200	1
		Repeat	633	300	1
High rep-rate	Test trials	Novel	567	600	5


		Novel	633	600	5
		Repeat	567	600	15
		Repeat	633	600	15
	Catch trials	Novel	567	1200	1


		Novel	633	300	1
		Repeat	567	1200	3
		Repeat	633	300	3

The sequence of events on each trial was as follows: fixation cross for 150 ms; black screen for a randomly selected interval between 300 and 400 ms; first face; black screen for 300 ms; second face; black screen for 2000 ms. Participants were instructed to make their response during the 2-s response window after the second face had disappeared.

Prior to the main task, participants completed eight practice trials on which the first image was shown for 600 ms and the second was shown for 300 ms (2 repetition trials and 2 non-repetition trials) or 1200 ms (2 repetition trials and 2 non-repetition trials); the practice trials used faces not used in the main task.

### Participants

Participants were recruited from the University of Essex participant pool and took part for payment. The final sample comprised 31 participants (12 male), ages 19–45 (*M* = 24.1, *SD* = 5.6). A further 4 were excluded, 3 because more than 10% of trials were excluded due to timing errors or missed responses, and 1 because she scored less than 80% correct on the catch trials. Of the final sample, 16 of the participants started with a low rep-rate block and 15 started with a high rep-rate block.

## Results

On average, participants failed to respond within the response window on 1.1% of trials.; trials with missing responses were excluded prior to analysis. The mean proportion correct for the catch trials was 96.8% (*SD* = 3.9%), indicating good engagement with the task.

The analysis focussed on performance on the test trials. The proportion of “longer” responses for each condition are reported in **Table 2** and were submitted to a 2 × 2 × 2 ANOVA with repetition rate (low vs. high), condition (repetition vs. non-repetition), and the relative duration of the second stimulus (shorter than first face vs. longer than first face) as within-subject factors. There was a main effect of duration: as one would expect, participants were more likely to respond “longer” when the second face was shown for more time than the first, *F*(1,30) = 42.15, *p* < 0.001, $\eta_{p}^{2}$ = 0.584. There was also a weak overall tendency for novel faces to be perceived as having longer duration than repeats, *F*(1, 30) = 4.99, *p* = 0.033, $\eta_{p}^{2}$ = 0.143. There was no main effect of rep-rate, *F*(1,30) = 0.887, *p* = 0.354, $\eta_{p}^{2}$ = 0.029, but rep-rate did modulate the effects of stimulus repetition, *F*(1,30) = 18.52, *p* < 0.001, $\eta_{p}^{2}$ = 0.382. Neither rep-rate nor trial type interacted with stimulus duration, and there was no three-way interaction [*F*(1,30) = 0.76, *p* = 0.390, $\eta_{p}^{2}$ = 0.025, *F*(1,30) = 3.01, *p* = 0.093, $\eta_{p}^{2}$ = 0.091, and *F*(1,30) = 1.54, *p* = 0.224, $\eta_{p}^{2}$ = 0.049, respectively].

**Table 2:** Mean proportions of trials on which the second face was judged to last longer than the first.

Rep-rate	Condition	Relative duration of target stimulus	*p* (“Longer”)
Low	Novel	Short	0.436 (0.139)
Low	Novel	Long	0.489 (0.156)
Low	Repeat	Short	0.339 (0.159)
Low	Repeat	Long	0.389 (0.187)
High	Novel	Short	0.388 (0.170)
High	Novel	Long	0.478 (0.179)
High	Repeat	Short	0.396 (0.163)
High	Repeat	Long	0.436 (0.154)


*Standard deviations are in parentheses. Rep-rate indicates the proportion of trials on which the second face was a repeat of the first (low = 25%, high = 75%); condition indicates whether the second face was novel or a repeat of the first. Relative duration indicates whether the second stimulus was shown for more or less time than the first*.

**FIGURE 1:**
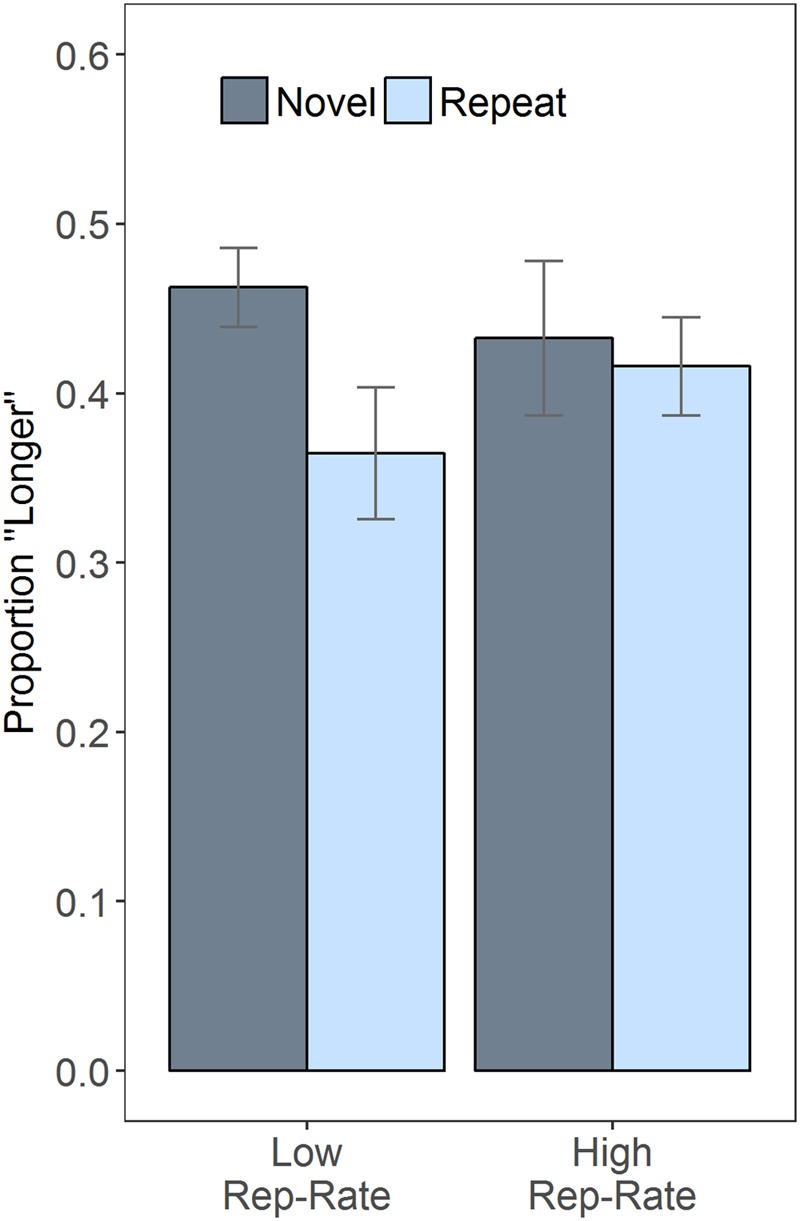
Repetition rate (rep-rate) modulates the effects of repetition. The plot shows the proportion of trials on which the second stimulus was judged longer than the first, as a function of rep-rate and whether the second stimulus was a repeat of the first or novel. Error bars are 95% confidence intervals, calculated as described in Morey (2008).

Given that duration did not modulate the effects of the other variables, it is helpful to plot the results after collapsing over duration (**Figure 1**), which clarifies the interaction between rep-rate and trial type: when repeats were rare, the novel faces were often judged as lasting longer than the repeats, an impression confirmed by a follow-up paired *t*-test, *t*(30) = 3.99, *p* < 0.001; when repeats were common, subjective duration was essentially unaffected by stimulus novelty, *t*(30) = 0.55, *p* = 0.585. **Figure 1** also indicates that, across conditions, there was a tendency to judge the second stimulus as being shorter than the first. This effect may have been due to the inclusion of long (1200 ms) comparison stimuli in some of the catch trials, which could have increased the reference level against which the target stimuli were judged (e.g., Morgan et al., 2000) – but this speculative suggestion awaits future empirical testing.

As an additional analysis (suggested by a Reviewer), we also examined the effects of repetition on sensitivity. Our design does not permit full psychometric curve fitting, so for each participant we computed the proportion of hits (when the second stimulus was correctly judged longer than the first) and false alarms (when the second stimulus was erroneously judged longer than the first) for each combination of repetition rate and condition, and used these to calculate d’. (In one instance the hit rate was zero and was replaced by 1/2N, where N is the number of trials in that condition). The resulting mean d’ values (with SDs in parentheses) were as follows: low rep-rate, novel stimuli: 0.143 (0.169); low rep-rate, repeats: 0.132 (0.316); high rep-rate, novel stimuli: 0.255 (0.358); high rep-rate, repeats: 0.110 (0.200). A 2 × 2 ANOVA indicated no main effect of rep-rate, *F*(1,30) = 1.02, *p* = 0.321, $\eta_{p}^{2}$ = 0.033, no main effect of repetition condition, *F*(1,30) = 3.06, *p* = 0.090, $\eta_{p}^{2}$ = 0.093, and no interaction, *F*(1,30) = 1.49, *p* = 0.231, $\eta_{p}^{2}$ = 0.047.

## Discussion

These results replicate and generalize the findings of Matthews (2015): novel stimuli were judged to last longer than repeated stimuli only when repeated stimuli were relatively rare (and hence presumably unexpected). When repetitions were common, there was no difference in the subjective duration of novel and repeated stimuli. The results from Matthews (2015) are therefore not limited to situations in which the to-be-compared stimuli have identical physical durations; indeed, the effects of repetition and repetition probability on subjective time were independent of the difference between the durations of the first and second stimuli, and we found no evidence that repetition or repetition rate affect the sensitivity of temporal discrimination.

Like the results of Matthews (2015), the current data indicate that the effects of repetition on subjective time are not due to the repeated stimuli being “predicted” or “expected.” Rather, the data suggest that first-order repetition compresses subjective time through some other mechanism, such as low-level sensory adaptation (Bruno et al., 2010; Cai et al., 2015), whereas expectation expands subjective duration (Birngruber et al., 2017), perhaps by improving the detection of the stimulus (Kim and McAuley, 2013) or the extraction of information from it (Matthews, 2015).

Several neuroimaging studies have found that repetition suppression is more pronounced when repeats are common than when they are rare (e.g., Summerfield et al., 2008, 2011; Andics et al., 2013). Under the coding efficiency account of time perception, the results of these imaging studies should be mirrored in subjective time (Pariyadath and Eagleman, 2007; Eagleman and Pariyadath, 2009); that is, the repetition effect on time perception should be more pronounced in high rep-rate blocks than in low rep-rate blocks, which is the opposite of the pattern found here. The present data therefore argue against a simple relationship between the macroscopic evoked response and the judgment of time (see also Benton and Redfern, 2016). Indeed, recent imaging studies suggest that the neural correlates of repetition and expectation are complex (e.g., Grotheer and Kovács, 2014, 2015), and are anatomically and temporally distinct (e.g., Todorovic and de Lange, 2012). The present data suggest that these dissociable patterns of activity have distinct functional consequences.

Our results can be accommodated within a broader theoretical framework recently proposed by Matthews and Meck (2016; Matthews and Gheorghiu, 2016). In this framework, which the authors label the *processing principle*, subjective time is a positive function of the strength of the percept, which refers to the ease with which information can be extracted from the stimulus and to the accompanying subjective vividness of the representation. Specifically, the processing principle posits that “the processes and variables that make a percept subjectively more vivid and objectively easier to identify, categorize, and evaluate also make it seem to last longer” (Matthews and Meck, 2016, p. 869). Perceptual strength, and therefore subjective duration, depends on the interplay of “bottom-up” and “top-down” processes. Under this account, the compressive effect of repetition is just one example of a variable that affects the effective strength of the sensory signal; other relevant factors include sensory adaptation (e.g., Johnston et al., 2006), modality (e.g., Jones et al., 2009), and salience (e.g., Matthews et al., 2011). Likewise, the expansive effects of higher-level expectations are mirrored by the effects of working memory (e.g., Pan and Luo, 2012), directed attention (e.g., Mattes and Ulrich, 1998), and long-term representations (e.g., Witherspoon and Allan, 1985).

The processing principle predicts that subjective duration is positively related to the ease with which information is extracted from a stimulus, but is agnostic about the cognitive or neural mechanisms that underlie time perception. Many different models of timing, including pacemaker-accumulator models (e.g., Zakay and Block, 1997), oscillator models (e.g., Gu et al., 2015), state-dependent network models (e.g., Buonomano et al., 2009), and drift-diffusion models (e.g., Simen et al., 2011), are all capable of accommodating the effects of repetition and expectation, but only by making *post hoc* assumptions about the effects of these factors on some aspect of the timing process. A key goal for future work will be the development of models in which the effects of bottom-up and top-down influences emerge from the model architecture, and which correspondingly generate new, testable predictions about how the interplay of these variables affects the perception of time.

## Author Contributions

WS designed the experiment. WS and AG prepared the stimuli and experiment. AG collected the data. WS and AG analyzed the data. WS wrote the paper.

## Conflict of Interest Statement

The authors declare that the research was conducted in the absence of any commercial or financial relationships that could be construed as a potential conflict of interest.
